# Mercury and psychosocial stress exposure interact to predict maternal diurnal cortisol during pregnancy

**DOI:** 10.1186/s12940-015-0016-9

**Published:** 2015-03-27

**Authors:** Hannah MC Schreier, Hsiao-Hsien Hsu, Chitra Amarasiriwardena, Brent A Coull, Lourdes Schnaas, Martha María Téllez-Rojo, Marcela Tamayo y Ortiz, Rosalind J Wright, Robert O Wright

**Affiliations:** Department of Pediatrics, Icahn School of Medicine at Mount Sinai, One Gustave L. Levy Place, Box 1198, New York, NY 10029 USA; Department of Preventive Medicine, Icahn School of Medicine at Mount Sinai, New York, NY USA; Department of Biostatistics, Harvard School of Public Health, Boston, MA USA; Department of Environmental Health, Harvard School of Public Health, Boston, MA USA; Division of Research on Public Health, National Institute of Perinatology, Mexico City, Mexico; Center for Evaluation Research and Surveys, National Institute of Public Health, Cuernavaca, Morelos Mexico; Center for Research in Nutrition and Health, National Institute of Public Health, Cuernavaca, Morelos Mexico

**Keywords:** Mercury, Psychosocial, Stress, Pregnancy, Cortisol, HPA axis

## Abstract

**Background:**

Disrupted maternal prenatal cortisol production influences offspring development. Factors influencing the hypothalamic-pituitary-adrenal axis include social (e.g., stressful life events) and physical/chemical (e.g., toxic metals) pollutants. Mercury (Hg) is a common contaminant of fish and exposure is widespread in the US. No prior study has examined the joint associations of stress and mercury with maternal cortisol profiles in pregnancy.

**Objectives:**

To investigate potential synergistic influences of prenatal stress and Hg exposures on diurnal cortisol in pregnant women.

**Methods:**

Analyses included 732 women (aged 27.4 ± 5.6 years) from a Mexico City pregnancy cohort. Participants collected saliva samples on two consecutive days (mean 19.52 ± 3.00 weeks gestation) and reported life stressors over the past 6 months. Hg was assessed in toe nail clippings collected during pregnancy.

**Results:**

There were no main effects of Hg or psychosocial stress exposure on diurnal cortisol (*p*s > .20) but strong evidence of interaction effects on cortisol slope (interaction B = .006, SE = .003, *p* = .034) and cortisol at times 1 and 2 (interaction B = -.071, SE = .028, *p* = .013; B = -.078, SE = .032, *p* = .014). Women above the median for Hg and psychosocial stress exposure experienced a blunted morning cortisol response compared to women exposed to higher stress but lower Hg levels.

**Conclusions:**

Social and physical environmental factors interact to alter aspects of maternal diurnal cortisol during pregnancy. Research focusing solely on either domain may miss synergistic influences with potentially important consequences to the offspring.

Cortisol is a hormone that is secreted by the adrenal cortex following activation of the hypothalamic-pituitary-adrenal (HPA) axis. Typically, cortisol secretion follows a diurnal pattern, such that cortisol levels are low overnight, begin to rise just before awakening, peak shortly after waking (~30-45 minutes), and subsequently decline throughout the day [1]. Cortisol plays a critical role in early development [2,3] and disruptions in prenatal maternal cortisol production may play a role in neurodevelopmental disorders, including impaired cognitive development, autism, and behavioral problems among offspring [4]. Consequently, understanding environmental contributors to altered maternal cortisol rhythms during pregnancy represents an important step towards preventing adverse outcomes in offspring.

Endocrine assessment, including glucocorticoid production, has been used in the detection of early or low-level effects of environmental stressors as a means of identifying individuals at risk for later health problems. The HPA axis is subject to potential disruption by both non-chemical (social stressors) and chemical factors [5-8].

Previous research has shown that both the experience of acute and chronic psychological stressors is associated with altered cortisol output [8,9]. In general, adverse experiences (i.e., stress) with a chronic or cumulative nature most strongly influence child health risk, perhaps by producing a more sustained (i.e., trait-like) shift in HPA axis functioning. Moreover, social environmental factors, including greater life stress and stress correlates (e.g., anxiety, depression), have been linked to altered diurnal cortisol production among pregnant women [10-12]. Stress-elicited physiological responses vary and are influenced by characteristics of the stressor (e.g., magnitude, timing, chronicity). Under chronic stress, the HPA axis may operate at higher or lower levels compared to normal homeostasis [8]. Due to the complexity of cortisol metabolism, disruptions of homeostasis may manifest in a variety of ways, such as lower cortisol at awakening, an altered morning rise, or a slower decline over the day. Each in turn has been linked with adverse health outcomes. For example, altered prenatal cortisol production in pregnant mothers, particularly elevated levels later in the day, has been associated with adverse child outcomes including low birth weight, poorer cognition, and repeated wheeze [13-15].

In parallel, a toxicology literature on cortisol disruption has developed. Exposure to toxic metals, such as mercury (Hg), is also being implicated in the alteration of HPA axis functioning [16] in animal studies on mammals and fish [17-20]. Hg is a heavy metal that, in organic form, e.g. methylmercury (MeHg), is readily absorbed by the gastrointestinal tract following ingestion [21]. Hg pollution in the environment is converted to MeHg by bacteria and because MeHg is poorly excreted, it bioaccumulates up the food chain with higher concentrations found in larger predatory animals, including predatory fish such as tuna, sharks and swordfish, and in the animals that consume them, such as humans [22]. Moreover, MeHg exposure has been linked to a number of adverse neurodevelopmental outcomes in humans, including reduced motor skills, neuromuscular changes, and a variety of cognitive function deficits [23,24]. MeHg exposure during pregnancy via maternal fish consumption has also been associated with adverse neurodevelopmental outcomes in their children, although not all studies have found deficits [25-27]. To date, no studies in humans have examined the relationship between prenatal Hg exposure and cortisol production in pregnant women.

Given that exposure to psychosocial stressors may lead to altered physiological states (e.g., greater inflammation, altered HPA axis functioning) that are similar to states induced by toxic chemicals, it is natural to speculate that joint exposure to both may increase an individual’s vulnerability to these external stressors in the physical environment. There has been an increasing call for the joint consideration of social and physical environmental influences on health [28-33]. Several animal studies have examined joint effects of prenatal stress and other metals (e.g., lead) on HPA axis functioning [5,34,35] and a few studies have examined effects of lead-stress interactions on the HPA axis in postnatal samples of older children [6,36]. To our knowledge, no study in humans has considered whether *combined* exposure to social stress and Hg may alter *diurnal* HPA axis functioning in humans synergistically.

In the present study we investigate potential synergistic influences of a social pollutant, negative life events, and a physical pollutant, Hg exposure, on diurnal daily cortisol rhythms in a sample of pregnant women participating in a Mexico City birth cohort. We hypothesize that the experience of negative life events and Hg exposure interact to alter maternal prenatal diurnal cortisol rhythms.

## Materials and methods

### Participants

Between 2007 and 2011, women who were at 12-24 weeks’ gestation and healthy, were recruited to participate in this study as part of an ongoing prospective Mexico City birth cohort. All women had to be at least 18 years of age, have access to a telephone, and plan to stay in Mexico City for at least three years following recruitment. Women with diagnoses of heart or kidney disease, who used steroid or antiepilepsy drugs, or who reported daily alcohol consumption were not eligible. There were 3898 women seen in the clinics over this time span, 84% of whom were eligible. Of these we enrolled 1054. All enrolled women signed a letter of informed consent in the late 1^st^ trimester and were seen for a study visit in the 2^nd^ trimester. Among the enrolled women the attrition rate was 30.6% due to women dropping out prior to delivery or having a spontaneous abortion (n = 93; 8.8%) or remaining enrolled but missing Hg data, psychosocial stress data, or cortisol data (n = 229; 21.7%) and being dropped from the analyses. These women did not differ from women who remained in the analyses with respect to socioeconomic status, body mass index, smoking during pregnancy, and parity (all *p*s > .10); they were marginally younger [t = -1.695, p = .090]. The final sample included 732 women (aged 27.4 ± 5.6 years). See Table 1 for a summary of participant characteristics. All participants provided written informed consent. This study was approved by the ethics boards of the Harvard School of Public Health, the Mexican National Institute of Public Health, the Mexican National Institute of Perinatology, and the Icahn School of Medicine at Mount Sinai.

**Table 1:** **Sample descriptives**

	**n (%)**	**M (± SD)**
*N* = 732		
Age (years)		27.4 (±5.6)
BMI		27.0 (±4.3)
Highest level of education		
Middle school or less	188 (25.7%)	
Technical post-middle school	107 (14.6%)	
High school or junior college	215 (29.4%)	
Technical post-junior college	32 (4.4%)	
College	175 (23.9%)	
Graduate	11 (1.5%)	
Psychosocial stress (CRISYS)		2.27 (±1.62)
Toenail mercury (μg/g)		0.19 (±0.15)
Gestational age at saliva collection		19.52 (±3.00)
Cortisol AUC (nmol/L; log)		13.93 (±2.48)
Cortisol slope (nmol/L; log)		−0.04 (±0.02)

BMI = body mass index; CRISYS = Crisis in Family Systems Scale; AUC = area under the curve;

### Mercury

Maternal Hg exposure was measured in toenail clippings obtained either during the 2^nd^ (n = 474, 64.8%) or 3^rd^ (n = 258, 35.2%) trimester of pregnancy. Total toenail Hg represents a longer term index of Hg exposure over several weeks to months and is largely reflective of MeHg [37]. Previous studies have shown toenail levels of Hg to be stable over time [38,39], to be highly correlated with Hg levels in blood and hair [39,40] and to be highly correlated with fish consumption [22,37,40].

We performed mercury assays using the Direct Mercury Analyzer 80 (Milestone Inc., Monroe, CT). This automatic mercury analyzer requires no sample digestion or pretreatment. The cleaned sample [41] of toenail was weighed into a nickel boat, thermally decomposed, amalgamated, and the released mercury measured by atomic absorption spectroscopy at 253.7 nm as a function of mercury concentration. Samples were analyzed by using an aqueous calibration curve. Quality control steps included daily calibration with verification of a high and low concentration standard for each working range, a procedural blank, and Certified Reference material GBW 07601 (human hair; Institute of Geophysical and Geochemical Exploration, Langfang, Hebei Province, People’s Republic of China). The detection limit (DL) for the mercury analysis was 0.5 x 10^−3^ μg. The DL for the sample varied according to sample weight. Sample weight varied from 0.007 g to 0.081 g and the DL varied from 0.006 μg/g to 0.077 μg/g (mean = 0.018 μg/g). Mercury recovery from the standard using this procedure was 86% - 120%, with intraday precision varying from 1.2% RSD to 9% RSD. Interday precision was 6.5%. Hg values in the present sample ranged from 0.0 – 1.9 μg/g.

### Psychosocial stress

Participants completed the Crisis in Family Systems [42] questionnaire which has been translated and validated in Spanish [43] to indicate stressful life events they had experienced over the past six months. The CRISYS questionnaire asks participants to indicate whether or not they experienced potentially stressful life events in 11 domains (financial; legal; career; relationships; safety in the home; safety in the community; medical issues pertaining to respondent; medical issues pertaining to close others; home issues; difficulties with authority; prejudice). In addition, participants indicated the valence of all experienced events, i.e. whether or not they perceived a certain event negatively or not. Participants were coded as having experienced any or no negative life events in each one of the 11 domains as done previously [44]. A sum score was created by counting the number of domains for which participants endorsed any negative events, resulting in possible scores from 0 (no negative events endorsed) to 11 (at least one negative event endorsed in each domain). In this sample, participants reported negative events in an average of 2.27 ± 1.6 domains (range 0-8). The CRISYS has previously been shown to have good test-retest reliability and to be a valid measure of life events [43]. It has furthermore been linked to health outcomes in a dose-response manner, such that exposure to negative life events in multiple domains is associated with worsening health outcomes [44], and has previously been linked to disrupted cortisol rhythms [45].

### Diurnal cortisol

All women collected saliva samples at home for the assessment of salivary cortisol. Saliva samples were collected when women were on average 19.52 ± 3.00 weeks pregnant. To capture the diurnal variation in cortisol production, participants were instructed to collect five saliva samples into tubes using the passive drool technique [46] on each of two consecutive days. Prior to sample collection, research staff instructed women to provide samples when they awoke (“when you open your eyes”), 45 minutes after waking, 4 hours after waking, 10 hours after waking, and at bedtime (“right before getting into bed”). Participants followed these instructions well, collecting their five saliva samples an average of .13 ± .28, .99 ± .40, 4.50 ± .74, 10.70 ± 1.20, and 15.21 ± 1.29 hours following waking. After collecting each sample, women recorded the collection time on the tube and in a diary. Participants were instructed not to eat, brush their teeth, or drink liquids for at least 15 minutes before providing a sample and not drink caffeinated beverages before collecting the first two samples. Women were asked to refrigerate samples until pickup on the third day after which they were stored at -70° C until shipment on dry ice to the laboratory of Dr. Clemens Kirschbaum in Dresden, Germany for assay. Samples were assayed in duplicates using a commercially available chemi-luminescence assay (IBL; Hamburg, Germany) with sensitivity of ~0.16 ng/ml. Intra- and interassay coefficients of variation were < 8%.

Cortisol values were log-transformed to reduce skewness. Total daily cortisol output, cortisol area under the curve (AUC), was computed by calculating the area under the curve statistic using the trapezoidal rule. Specifically, for each day and each participant a line depicting cortisol at each of the five collection time points was plotted, and AUC calculated as the sum of the four trapezoids below that line. AUCs of both days were averaged. A higher AUC is suggestive of greater overall cortisol output over the course of the day. In addition, cortisol values were averaged across both collection days to increase stability and cortisol slope (cortisol values/corresponding time since wake-up) computed. Steeper slopes indicate a more rapidly declining cortisol output throughout the course of the day, flatter slopes indicate a slower decline in cortisol output over the course of the day.

### Potential confounders

Participating women reported on demographic information at the time of recruitment, including age and highest level of education. Body mass index (BMI) was computed as kg/m^2^ based on weight and height measured by a trained research assistant during a second trimester visit. All analyses also controlled for the trimester during which toe nail clippings for Hg assessment were collected and for weeks of gestation at the time of saliva sample collection, as cortisol levels are known to rise over the course of pregnancy [47].

### Statistical analyses

**Figure 1:**
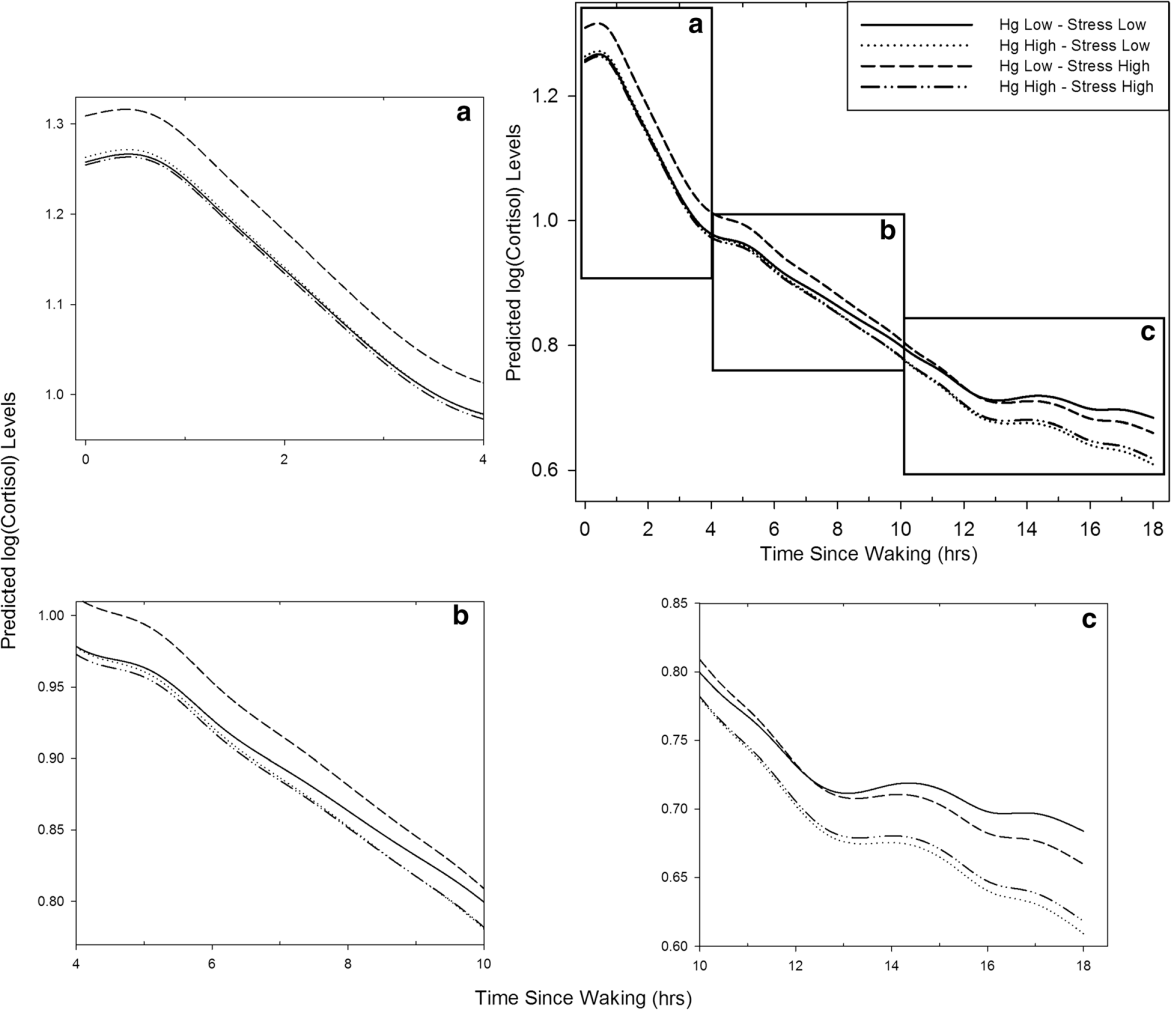
**Maternal Hg and psychosocial stress exposure interact to influence maternal diurnal cortisol profiles during pregnancy: predicted values of Log(cortisol) over time since waking(hours) from functional mixed model with penalized splines** ***.*** The full figure depicts predicted cortisol values across the entire day; panels **a)**, **b)** and **c)** depict close-ups of predicted cortisol values in the morning (0-4 hours following waking), mid-day (4-10 hours following waking), and in the evening (10-15 hours following waking), respectively. Functional mixed models with penalized splines were used to estimate main effects of psychosocial stress and Hg exposure and interactions of the same on the mean cortisol curve for a given individual. Participants were placed into one of the four groups depicted based on whether they scored above or below the median for psychosocial stress and Hg exposure.

Eleven Hg and three CRISYS scores were more than three standard deviations from the mean. Winsorization to reduce these scores to the next highest score did not change our results, however, and consequently the original values were retained for the analyses reported below. We first tested the main effects of Hg exposure and exposure to negative life events (controlling for the other) on maternal diurnal cortisol profiles (area under the curve, diurnal slope, and cortisol levels at individual collection time points averaged across both days and controlling for time since waking) using multiple linear regression analyses. Next, we used multiple linear regression analyses to test whether Hg exposure interacted with exposure to life stressors to predict maternal diurnal cortisol profiles. Following the guidelines for interaction effects provided by Aiken and West [48] Hg and life stress variables were centered and subsequently multiplied to create the interaction term. We first entered Hg exposure, negative life events, and covariates into the model, then added the interaction term between Hg exposure and negative life events to examine the effect modification. Regression analyses were conducted using SPSS Statistics 20 for Windows (IBM). In addition, to visualize and enhance the interpretability of our results, treating the five log(cortisol) measures as observations from an overall nonlinear curve for each individual, we used functional mixed models with penalized splines (FMPS, [49]) using R [50] to estimate main effects of and interactions between the two exposures of interest on the mean cortisol curve for a given individual. Participants were placed into one of 4 groups indicating whether they were below the median on both Hg exposure and life stress (LL), below the median for Hg exposure and above the median for life stress (LH), above the median for Hg exposure and below the median for life stress (HL), or above the median for both Hg exposure and life stress (HH). Time since waking was used as the time metric for each individual. Predicted diurnal cortisol profile (log(cortisol) over time (hours)) using FMPS for each group was plotted in Figure 1.

## Results

### Main effects of Hg exposure and psychosocial stress

First, we investigated the main effects of Hg exposure and life stress on maternal diurnal cortisol profiles. When used to predict maternal diurnal cortisol outcomes independently, Hg exposure was unrelated to cortisol AUC (*p* > .30) and diurnal cortisol slope (*p* > .30). We also assessed the influence of Hg exposure on cortisol values at the individual times of data collection, averaged across the two days, controlling for time since waking. Maternal Hg exposure was unrelated to cortisol at the first four collection times over the course of the day (all *p*s > .50) but marginally associated with lower cortisol at time 5, i.e. before bedtime (B = -.080, SE = .044, *p* = .070).

Exposure to stressful life events, when Hg is also entered into the model, was also unrelated to cortisol AUC (*p* > .60) and diurnal slope (*p* > .60). Report of more stressful life events was associated with marginally higher cortisol at the first collection time of the day, i.e. immediately following waking (B = .007, SE = .004, p = .075). Exposure to stressful life events was unrelated to cortisol levels at all other collection times throughout the day (all *p*s > .10).

See Table 2 for details regarding our main effects analyses.

**Table 2:** **Regression analyses of main and interaction effects between Hg and psychosocial stress exposure**

**Outcome**	**Model**	**Psychosocial stress (CRISYS)**	**Hg**	**CRISYS x Hg**
		**[B (SE, p)]**	**[B (SE, p)]**	**[B (SE, p)]**
AUC	Main effects	.026 (.057, .649)	-.536 (.596, .369)	-
	Interaction effects	.027 (.057, .640)	-.640 (.606, .292)	-.400 (.420, .341)
Slope	Main effects	.000 (.000, .622)	-.004 (.004, .304)	-
	Interaction effects	.000 (.000, .604)	-.002 (.004, .529)	**.006 (.003, .033)**
Time 1	Main effects	.007 (.004, .075)	-.010 (.041, .798)	-
	Interaction effects	.007 (.004, .070)	-.029 (.041, .481)	**-.071 (.028, .013)**
Time 2	Main effects	.006 (.004, .198)	.026 (.046, .561)	-
	Interaction effects	.006 (.004, .188)	.006 (.046, .898)	**-.078 (.032, .014)**
Time 3	Main effects	.003 (.004, .451)	-.024 (.041, .558)	-
	Interaction effects	.003 (.004, .446)	-.033 (.042, .432)	-.033 (.029, .251)
Time 4	Main effects	.002 (.004, .632)	-.028 (.045, .535)	-
	Interaction effects	.002 (.004, .634)	-.026 (.045, .569)	.007 (.031, .822)
Time 5	Main effects	.002 (.004, .620)	-.080 (.044, .070)	-
	Interaction effects	.002 (.004, .624)	-.077 (.045, .086)	.011 (.031, .721)

AUC = area under the curve; Hg = mercury; CRISYS = Crisis in Family Systems Scale;

Note: All analyses controlled for maternal age, education, body mass index, weeks gestation at the time of saliva collection, and the pregnancy trimester during which toe nails were collected for Hg assessment. Analyses investigating the influence of Hg and psychosocial stress on cortisol levels at individual time points additionally controlled for time since waking. Significant findings are highlighted in bold.

### Interaction effects of Hg and psychosocial stress

**Figure 2:**
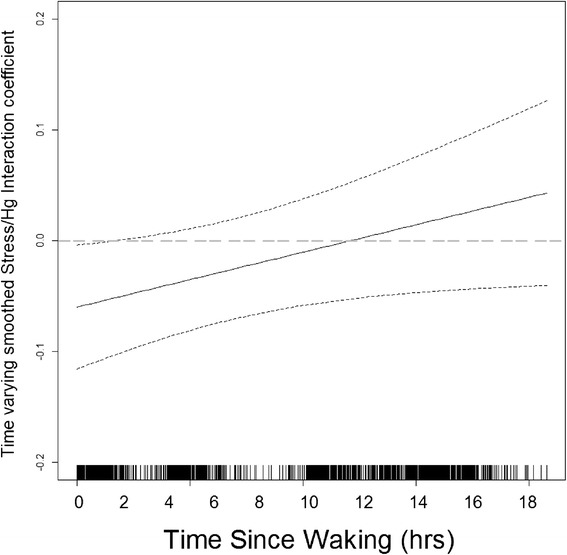
**Maternal Hg and psychosocial stress exposure interact to influence maternal diurnal Cortisol profiles during pregnancy: time-varying interaction beta.** The graph of the time-varying interaction beta shows a significant, negative interaction between Hg and psychosocial stress exposure during the first two hours of the day. Early morning differences in cortisol (during the first two hours following waking) result in flatter slopes among women exposed to greater psychosocial stress and Hg levels compared to women exposed to greater psychosocial stress but lower levels of Hg.

Second, we investigated whether exposure to negative life events and Hg exposure interacted to influence maternal diurnal cortisol. There was a significant Hg x negative life events interaction effect on maternal cortisol slope (interaction B = .006, SE = .003, *p* = .033) but not maternal cortisol AUC (*p* > .30). As can be seen in Figure 1 (panel a), there is no effect of Hg exposure on cortisol in the low stress group; however, in the high stress group, there is a negative effect of Hg exposure such that the stress response (increased cortisol) is blunted among women exposed to Hg levels above the median. Conversely, women in the high stress group who are below the median for Hg exposure show evidence of greater cortisol levels in the morning. Panels b) and c) of Figure 1 show that this negative effect of Hg exposure among the higher stress group weakens later in the day. When considering the Hg x life stress interaction at individual cortisol collection time points throughout the day, there also were significant negative effects at time 1, i.e. immediately following waking (interaction B = -.071, SE = .028, p = .013), and time 2, i.e. approximately one hour following waking (interaction B = -.078, SE = .032, p = .014); see Table 2. Cortisol levels at later times of the day were not significantly associated with the Hg x life stress interaction term (all *p*s > .20). Figure 2, a graphical depiction of the results from FMPS, shows that the interaction effect is significant during the first two hours following waking, further suggesting that it is the early morning differences that affect the diurnal slope (flatter among women exposed to greater psychosocial stress and Hg levels compared to women exposed to greater psychosocial stress but lower levels of Hg).

## Discussion

This study is among the first to examine the synergistic influence of toxic exposures in the physical environment, i.e. Hg, and in the social environment, i.e. negative life events, on human HPA axis activity. In addition, in the present study we focused on examining these associations among pregnant women, as cortisol production during pregnancy is increasingly being linked to important developmental outcomes among offspring [2,4]. Environmental toxicants with the potential to alter maternal cortisol output during pregnancy hence may have important consequences for the healthy development of offspring. Toxicants that commonly occur jointly with stressful life events need to be better understood. Our findings highlight the importance of considering physical and social environmental influences jointly, rather than independently, as their synergistic effects may have a stronger influence on physiological outcomes of interest, including HPA activity in the present study. In addition, with regards to health disparities, the joint occurrence of stressful life events and toxic chemicals may be most common in economically disadvantaged populations.

As part of the present study we examined both main effects and interaction effects of Hg and psychosocial stress exposure. We found that, in a main effects model, maternal prenatal Hg exposure was marginally negatively associated with cortisol levels before bedtime (though not at other times). We found no main effects of negative life events, although exposure to more negative life events was associated with marginally higher cortisol levels in the morning. These findings are in line with previous research that has linked the experience of greater stress to a greater increase in cortisol following waking [51] and recent research linking even low-level Hg exposure among children to a blunted diurnal cortisol response to stress [52]. When considering synergistic influences of the social and physical environment we found that among women exposed to high levels of psychosocial stress (more negative life events), simultaneous exposure to greater Hg levels resulted in a blunted cortisol response early in the day compared to women experiencing greater psychosocial stress but lower levels of Hg exposure.

These findings are also in line with limited evidence from animal studies suggesting that Hg exposure can disrupt HPA functioning, leading to, for example, reduced cortisol levels in catfish [53]. A small number of studies investigating the effect of Hg exposure among human adults have found no impact of Hg exposure on HPA axis functioning [54,55]; however, these studies are limited by their small sample sizes and the indices of HPA axis functioning used, specifically, one-time assessments of serum cortisol levels which do not allow for the evaluation of daily cortisol rhythms. Existing research in fish further supports the idea that psychosocial stress and Hg exposure interact. Miller et al. [19] found that when treating lake trout thymocytes with either Hg alone or Hg and cortisol together, the presence of cortisol increased the toxicity of Hg. In addition, one study investigating yellow perch and northern pike provides further evidence of the potential synergistic effects of Hg exposure and stress. Hontela et al. [56] found that fish from polluted sites (including, but not restricted to, Hg pollution) failed to show the typical cortisol response to capture that was observed in control fish from unpolluted areas, again suggesting blunted cortisol responses in response to Hg exposure.

Strengths of the present study include the large sample size relative to nearly all other reports of chemical exposure on cortisol as well as the collection of saliva samples across five time points on each of two days, allowing for more stable assessments of daily cortisol rhythms. In addition, we were able to assess Hg levels in toe nail clippings collected during pregnancy, providing us with a measure of longer-term Hg exposure across several months. Our population is not coastal and is not high in fish consumption, thus it is interesting that we see an association between Hg exposure and diurnal cortisol patterns at these relatively low levels of Hg exposure, suggesting that our findings may be conservative. Future studies should investigate these associations among populations exposed to higher levels of Hg.

Although we were able to evaluate the association of simultaneous exposure to Hg and negative life events and diurnal cortisol slope, an indicator of the diurnal rhythm of cortisol production, it is possible that Hg exposure and exposure to negative life events might be differentially associated with cortisol patterns in response to acute stressors. Future research should investigate whether acute stress responses are altered as a function of these exposures. Similarly, the present study employed a summary score representing different types of psychosocial stressors experienced over a 6-month period. It is unclear whether certain types of stressors, e.g. stressors directly impacting home rather than work life, are more likely to interact with Hg and result in potentially adverse physiological outcomes. Due to sample size restrictions we were not able to investigate interaction effects with individual negative life stress domains as part of the present study. However, as previously mentioned, existing research suggests a dose-response relation between exposure to negative life events across multiple domains and adverse health outcomes [44] and has linked the CRISYS to altered cortisol rhythms [45]. As such exposure may place increased burden on individuals and result in greater distress, considering cumulative stress exposure may be valuable. It is also important to note that this study was not experimental and that, consequently, alternative explanations for our findings cannot be ruled out. For example, the exact effects of Hg exposure on human HPA-axis functioning is not clearly understood, nor are the possible effects of diurnal cortisol on Hg toxicokinetics. Hence, our findings must be viewed with caution until the underlying mechanisms linking Hg and cortisol in humans are better understood. In addition, we were only able to measure total Hg in the present study. Although total Hg has been shown to be largely reflective of MeHg [37], it cannot be ruled out that our findings are the result of an interaction between exposure to psychosocial stressors and levels of inorganic Hg and this needs to be investigated further. Due to the use of an entirely Mexican birth cohort, the generalizability of the present findings are also limited. However, both the levels of Hg found in toe nails and the scores on the CRISYS questionnaire are comparable to those found in American samples [57], suggesting that similar associations may exist among non-Mexican populations as well. Finally, future studies should also examine the combined impact of maternal Hg exposure and life stress during pregnancy on relevant developmental outcomes among their offspring, such as birth weight and indicators of cognitive development.

## Conclusion

This study suggests that physical and social environmental influences interact to alter aspects of maternal diurnal cortisol profiles, an indicator of HPA axis activity, among a sample of pregnant women in Mexico City. Specifically, among women exposed to more negative life events, exposure to higher levels of Hg resulted in a blunted salivary cortisol response early in the day. With respect to research focusing on environmental effects on pregnancy outcomes or developmental effects among offspring, these findings suggest that conducting research focusing solely on either physical or social pollutants may miss important synergistic associations that have the potential to negatively impact the healthy development of offspring.

## Abbreviations

BMI: Body mass index
Hg: Mercury
HPA: Hypothalamic-pituitary-adrenal
MeHg: Methylmercury
